# Psychological distress in medical patients 30 days following an emergency department admission: results from a prospective, observational study

**DOI:** 10.1186/s12873-016-0097-y

**Published:** 2016-08-24

**Authors:** Lukas Faessler, Alexander Kutz, Sebastian Haubitz, Beat Mueller, Pasqualina Perrig-Chiello, Philipp Schuetz

**Affiliations:** 1 Institute of Psychology, University of Berne, Berne, Switzerland; 2 University Department of Internal Medicine, Kantonsspital Aarau, Tellstrasse, 5001 Aarau, Switzerland

**Keywords:** Emergency patients, Psychological distress, Risk factors

## Abstract

**Background:**

Psychological distress in medical patients admitted to the emergency department (ED) is not well studied. Our aim was to investigate the extent of psychological distress in a broad and unselected medical patient sample 30 days after ED admission and its association with socio-demographic and clinical variables.

**Method:**

We used data from a prospective observational cohort study including 1575 consecutive adult medical patients presenting to the ED with acute somatic conditions. Outcome variables were patient’s psychological distress measured by the 4-item Patient Health Questionnaire (PHQ–4) and self-rated health assessed 30 days after ED admission using telephone interviews. Risk factors included socio-demographic variables (e.g. gender, marital status), clinical presentation (e.g. illness severity, main initial diagnosis) and course of illness (e.g. rehospitalisation, length of hospital stay).

**Results:**

A total of 38 % of patients had evidence for psychological distress 30 days after ED admission. Multivariate analysis found female gender (adjusted odds ratio [aOR] 1.35, 95 % confidence interval [CI] 1.02 to 1.78), comorbid psychiatric disorder (aOR 1.63, 95 % CI 1.08 to 2.62), discharge to a post-acute care institution (aOR 1.47, 95 % CI 1.03 to 2.09), unplanned rehospitalisation (aOR 2.38, 95 % CI 1.47 to 3.86), and unplanned visit at general practitioner (aOR 4.75, 95 % CI 2.57 to 8.80) to be associated with distress at day 30 following ED admission.

**Conclusions:**

One month after ED admission a significant number of patients still show a moderate amount of psychophysical distress. Strongest related variables were course of illness, in particular unplanned general practitioner visits. Future interventional studies should assess possibilities to reduce distress in patients at increased risk.

**Trial registration:**

NCT01768494, January 9, 2013 (registration date), February 25, 2013 (enrolment of first participant).

## Background

Most patients presenting to an emergency department (ED) with acute somatic conditions are likely to experience this situation as stressful and associated with considerable psychological distress. Although there is no well accepted definition for psychological distress, it generally involves a diversity of affective responses such as fear, hopelessness, sadness, anxiety and frustration [1]. Prior studies have associated psychological distress mainly with the presence of depressive and anxiety symptoms. Therefore, it can be defined as a psychological state or a clinical syndrome. There is an ongoing debate in the literature in terms of the conceptualization of psychological distress [2, 3]. The prevalence rates of severe psychological distress (high or very high distress) in patients admitted to the ED are reported between 34 and 47 % [4, 5]. While most patients may cope well with the challenge of an acute physical illness [6], some may still suffer from persisting psychological distress after ED and hospital discharge. To our knowledge, only one study investigated psychological distress post admission to the ED. Shah and colleagues [7] evaluated patients in the ED and 14 days thereafter for depression and cognitive impairment. Two-weeks following ED admission, they found 6 % of older medical patients to have depression. Another study reported 12 % of patients had anxiety and 22 % had depressive symptoms one year after hospital admission [8]. Also, studies found a high percentage of patients to have psychiatric symptoms after hospital discharge [9–12].

Several factors may play a role in patients’ psychological adaptation to an ED admission such as demographic characteristics (e.g. age, gender), clinical presentation at ED (e.g. diagnosis, severity of disease) and the illness trajectory (e.g. rehospitalisation, length of hospital stay). These possible risk factors for an adverse psychological outcome following ED admission are still not well understood. Using the Global Assessment of Functioning (GAF) and the Karnofsky Performance Status Scale (KPS), McKenzie and colleagues [12] found lower initial psychological and physical functioning in hospitalized medical patients with persistence of depressive and anxiety disorders 3 months following discharge. Other studies found that physically ill patients with a positive screening for a depressive disorder or a major depression as assessed by the DSM-IV showed a longer hospital stay [13, 14]. However, most of these studies did not control for confounding variables such as main diagnosis and comorbidity. Furthermore, the severity of a medical disease or having a diagnosis of a chronic illness were related with psychological distress [15]. Additionally, evidence suggests that prevalence rates of depressive symptoms varies among different medical diagnoses and the specialties concerned with treating them with highest rates in inpatients from the neurology department (32.1 %) followed by gastroenterology (25.5 %), cardiology (22.9 %), nephrology (20 %) and respiratory department (7.1 %) [16]. Also, female patients showed more psychological symptoms after hospital discharge compared to males [9]. Depressive symptoms were higher in patients with a lower family income or with a marital status such as divorced, widowed or separated [15, 16]. Most of these studies were limited to small sample sizes and a narrow focus on specific patient populations rather than broader patient populations. Beyond psychological consequences, a patient’s own self-rated health assessment may be adversely affected by an acute somatic condition leading up to an ED admission. Studies have shown that patients presenting to the ED report poor health quality [17, 18]. Chin and colleagues found worsening assessments of self-rated health during acute illness compared to baseline levels prior to ED admission [17].

Little is known about prospective studies assessing the extent of psychological distress present in patients following ED admission. Furthermore, there is a lack of prospective studies assessing the predictors of psychological distress and self-rated health in patients following ED admission. Our aim was to determine the proportion of medical patients identified as psychologically distressed defined by the 4-item Patient Health Questionnaire (PHQ–4) and to assess their self-reported state of health 30 days following ED discharge. We also assessed the relationship between demographic and medical variables (clinical presentation and course of illness) with psychological distress and self-reported health.

## Methods

### Study design and setting

We prospectively included consecutive adult medical ED patients (age ≥ 18) presenting between February 25 and September 24, 2013. After initial ED examination, patients were included in a quality control registry with a 30 day follow-up telephone interview (TRIAGE study) [19].

This study was registered at the ‘ClinicalTrials.gov’ registration website (http://www.clinicaltrials.gov/ct2/show/NCT01768494). In view of the observational quality control nature of the study, the Institutional Review Board (IRB) of the Canton of Aargau approved the study and waived the need for informed consent (EK 2012/059).

Data regarding the two outcome variables of psychological distress and reported health state were collected 30 days after ED admission using the telephone interview. In addition, for these outcome variables we analyzed correlates related to demographic characteristics, clinical presentation and course of illness. Data in terms of demographic characteristics included age, gender, marital, and health insurance status. Clinical presentation consisted of diagnosis (e.g. cardiovascular, pulmonary or infectious diseases), co-existing illnesses (e.g. diabetes or hypertension), disease severity, and treatment priority. Data in terms of demographic characteristics, clinical presentation at ED admission, any transfers to an intensive care unit (ICU), the location after hospital discharge and the number of length of index hospital stay were assessed prospectively until hospital discharge using the routinely gathered information from the hospital electronic medical system. Information about the course of illness was taken from the 30 days follow-up Interview. These variables included unplanned rehospitalisation, unplanned readmission to the ED and unplanned visit at general practitioner (GP).

### Measurements

For the assessment of psychological distress, the PHQ–4 was used as an ultra-brief screening tool to assess psychological distress [20]. The PHQ-4 consists of two subscales, namely anxiety and depression. The four items are introduced with the question: “Over the last 2 weeks, how often have you been bothered by the following problems?” Response options are “not at all”, “several days”, “more than half the days”, and “nearly every day”, scored as 0, 1, 2, and 3, respectively. Reliability and validity of PHQ-4 scale has been previously reported [21].

Patient’s self-rated health was assessed using the EuroQol visual analogue scale (VAS) for rating of own-health state and the common core of different domains of health states [22]. This scale requires respondents to rate their composite health state on a 0–100 % thermometer scale (*0* = worst imaginable health state, *100* = best imaginable health state). The responses were found to be very reliable. In patients with dementia and/or cognitive impairment, the scores were used as reported by the patients.

The Manchester triage system (MTS) [23] was used to estimate the treatment priority. The MTS assigns patients to one of 52 flowchart diagrams based on the initial presenting complaint. For each of these diagrams red flags are defined based on the clinical presentation and/or vital signs. A triage nurse categorizes patients into different algorithms, and determines urgency of treatment using different urgency levels (*immediate* = demand immediate medical evaluation, *very urgent* = need evaluation within 10 min., *urgent* = assessment within 30 min., *standard* = evaluation within 90 min. and *non-urgent* = patient can wait for assessment for up to 120 min.). For the purpose of this analysis, the MTS was divided into two categories: urgent (targeting waiting time up to 10 min) and non-urgent. Furthermore, disease severity was estimated by the number of acute medical problems upon ED discharge by the treating emergency physician team.

All patients were contacted 30 days after hospital admission with a telephone interview using a predefined questionnaire to assess clinical course. Patients were asked if they had an unplanned rehospitalisation, an unplanned readmission to the ED or an unplanned visit at the GP in the time period between discharge from the ED and the telephone interview. Furthermore, patients were asked whether they were discharged to home or to a post-care institution (e.g. nursing home).

### Statistical analysis

The overall psychological distress (PHQ-4) score was found to be asymmetrically distributed and negatively skewed (mean = 1.4, SD = 2.5, range = 0 – 12). Therefore, we dichotomized the PHQ-4 score into two groups of patients, one without psychological distress (PHQ-4 score of zero) and the other with psychological distress (PHQ-4 score between 1 and 12). Self-rated health was treated as a continuous parameter. This outcome variable was not found to be normally distributed. We therefore used, as suggested by Acock [24], bootstrap estimation of the standard errors including several random samples with replacement.

Discrete variables are expressed as counts (%) and continuous variables are expressed as medians and interquartile ranges (IQR) unless stated otherwise. For estimation of univariate relationships between demographic characteristics, clinical presentation, and course of illness and the outcome variables, logistic and linear regression analyses were used. All testing was two-tailed and *p*-values less than 0.05 were included in a multiple regression analysis to determine outcome. Analyses were performed using Stata 12.1 (StataCorp LP, College Station, Texas, USA).

## Results

### Study population and baseline characteristics

Out of a total of 1863 initially included patients, 288 patients were excluded (118 patients died within 30 days, 15 patients were lost to follow-up, 134 patients declined to be interviewed and 21 patients had other reasons such as insufficient German skills, were medically unstable or had cognitive impairment). Thus, the final study sample comprised 1575 medical ED patients.

The sample description is presented in Table 1. Median age of the sample was 68 years with 59 % males. The majority of the patients were married (62 %) and had general health insurance (78 %). The most frequent main initial diagnoses were cardiovascular diseases (26 %), neurological disorders (23 %) and infectious diseases (16 %). Patients had a high burden of comorbidities including chronic renal failure (26 %), hypertension (14 %), cancer (14 %) and stroke (14 %). At ED admission, most of the patients had a high treatment priority (70 %) and had a median of 2 acute medical problems. During hospital stay, 95 (6 %) patients were transferred to an ICU, 116 (7 %) were re-hospitalised, 61 (4 %) were re-admitted to the ED and 54 (3 %) needed to be seen by a GP within 30 days. The median length of hospital stay was 5 days. The majority of the patients (81 %) were discharged home and the median psychological distress was 0 with an interquartile range between 0 and 2.

**Table 1:** Sample description

*N* = 1575	*n* (%)
Demographic variables	
Age, median (IQR)	60 (56–78)
Gender	
Female	647 (41.2 %)
Male	924 (58.8 %)
Marital status	
Divorced/Separated	171 (11.1 %)
Single	181 (11.8 %)
Married	953 (62.0 %)
Widowed	231 (15.0 %)
Health insurance^a^	
Basic	1190 (77.7 %)
Half private	230 (15.0 %)
Private	111 (7.3 %)
Initial clinical presentation	
Main initial diagnosis	
Infectious disease	233 (15.8 %)
Cancer	85 (5.8 %)
Immune disorder	25 (1.7 %)
Metabolic disorder	29 (2.0 %)
Psychiatric disorder (incl. intoxication)	35 (2.4 %)
Neurological disorder	343 (23.3 %)
Cardiovascular disease	385 (26.1 %)
Pulmonary disease	69 (4.7 %)
Digestive tract disease	135 (9.2 %)
Musculoskeletal disorder	74 (5.0 %)
Miscellaneous	60 (4.1 %)
Comorbidity	
Hypertension	181 (14.3 %)
Chronic heart failure	27 (2.1 %)
Coronary heart disease	103 (8.1 %)
Chronic obstructive pulmonary disease	32 (2.5 %)
Dementia	21 (1.7 %)
Diabetes	125 (9.9 %)
Stroke	176 (13.9 %)
Psychiatric disorder	140 (8.9 %)
Toxic	96 (7.6 %)
Cancer	178 (14.1 %)
Renal failure	327 (25.8 %)
Triage priority	
Urgent	571 (70.1 %)
Non-urgent	244 (29.9 %)
Number of acute medical problems, median (IQR)	2 (1–4)
Course of illness	
Rehospitalisation	116 (7.4 %)
Readmission ED	61 (3.9 %)
Unplanned GP visit	54 (3.4 %)
Intensive Care Unit	95 (6.0 %)
Location after discharge	
Home	1239 (81.0 %)
Post-care institution (e.g. nursing home)	291 (19.0 %)
Length of hospital stay (days), median (IQR)	5 (3–8)
Outcome variables	
Psychological distress, median (IQR)	0 (0–2)
No distress	978 (62.1 %)
Distress	597 (37.9 %)
Subjective health state, median (IQR)	80 (60–90)

*ED* Emergency department, *GP* General practitioner

^a^In Switzerland, the healthcare system is a combination of public, subsided private and totally private systems. Every Swiss resident is obliged to have basic health and accident insurance. Many people top up the basic cover with supplementary private health insurance

### Identification and correlates of psychological distress and self-rated health

About 38 % of patients met our definition of being psychologically distressed 30 days after ED admission. In addition, the median for the EuroQuol at this point was 80 % (IQR 60 to 90 %).

Significant associations between demographic characteristics, clinical presentation, and course of illness and psychological distress are shown in Table 2. Psychological distress was associated with female gender, unmarried status, psychiatric or musculoskeletal disorders, comorbidity with chronic obstructive disease, psychiatric disorders, intoxication, number of acute medical problems, rehospitalisation, readmission to the ED, unplanned GP visit, discharge to a post-acute care institution and length of hospital stay. These variables were further evaluated in a multiple logistic regression analysis and showed a likelihood-ratio chi-squared (13) of 68.39, *p* < 0.001.

**Table 2:** Regression analyses of significant risk factors related to patients psychological distress 30 days after ED presentation

		Logistic regression				Multiple logistic regression^a^			
	*N* (n)	OR	95 % CI		*p*-value	OR	95 % CI		*p*-value
Socio-demographic variables									
Gender (1 = female)	1571 (647)	1.36	1.10	1.67	0.004**	1.35	1.02	1.78	0.035*
Marital status (1 = not married)	1536 (583)	1.43	1.16	1.77	0.001**	1.23	0.93	1.63	0.150
Initial clinical presentation									
Main diagnosis	1473								
Psychiatric disorder (inlc. intoxication)^b^	35	2.23	1.13	4.38	0.021*	1.37	0.53	3.57	0.521
Musculoskeletal disorder^b^	74	1.68	1.05	2.68	0.030*	1.04	0.56	1.95	0.901
Comorbidity	1266								
COPD^c^	32	1.53	1.03	2.28	0.035*	1.05	0.62	1.77	0.856
Psychiatric disorder^c^	140	2.10	1.45	3.05	<0.001***	1.63	1.08	2.62	0.021*
Intoxication^c^	96	1.51	1.04	2.19	0.029*	1.28	0.74	2.24	0.380
Number of acute medical problems	1409	1.09	1.02	1.17	0.007**	1.03	0.95	1.12	0.479
Course of illness									
Rehospitalisation (1 = yes)	1575 (116)	2.06	1.41	3.01	<0.001***	2.38	1.47	3.86	<0.001***
Readmission ED (1 = yes)	1575 (61)	1.73	1.04	2.89	0.036*	0.78	0.39	1.57	0.483
Unplanned visit at GP	1575 (54)	3.74	2.09	6.71	<0.001***	4.75	2.57	8.80	<0.001***
Discharge post-care institution (1 = yes)	1530 (291)	1.91	1.47	2.47	<0.001***	1.47	1.03	2.09	0.033*
Length of hospital stay	1239	1.04	1.02	1.06	<0.001***	1.02	0.99	1.04	0.149

*p* < 0.05*, *p* < 0.01**, *p* < 0.001***

^a^pseudoR^2^ = 0.0488 (*N* = 1344)

^b^all other diagnoses were used as dummy variables

^c^all other comorbidities were used as dummy variables

*OR* Odds ratio, *CI* Confidence interval, *COPD* Chronic obstructive pulmonary disease, *ED* Emergency department, *GP* General practitioner

**Fig. 1:**
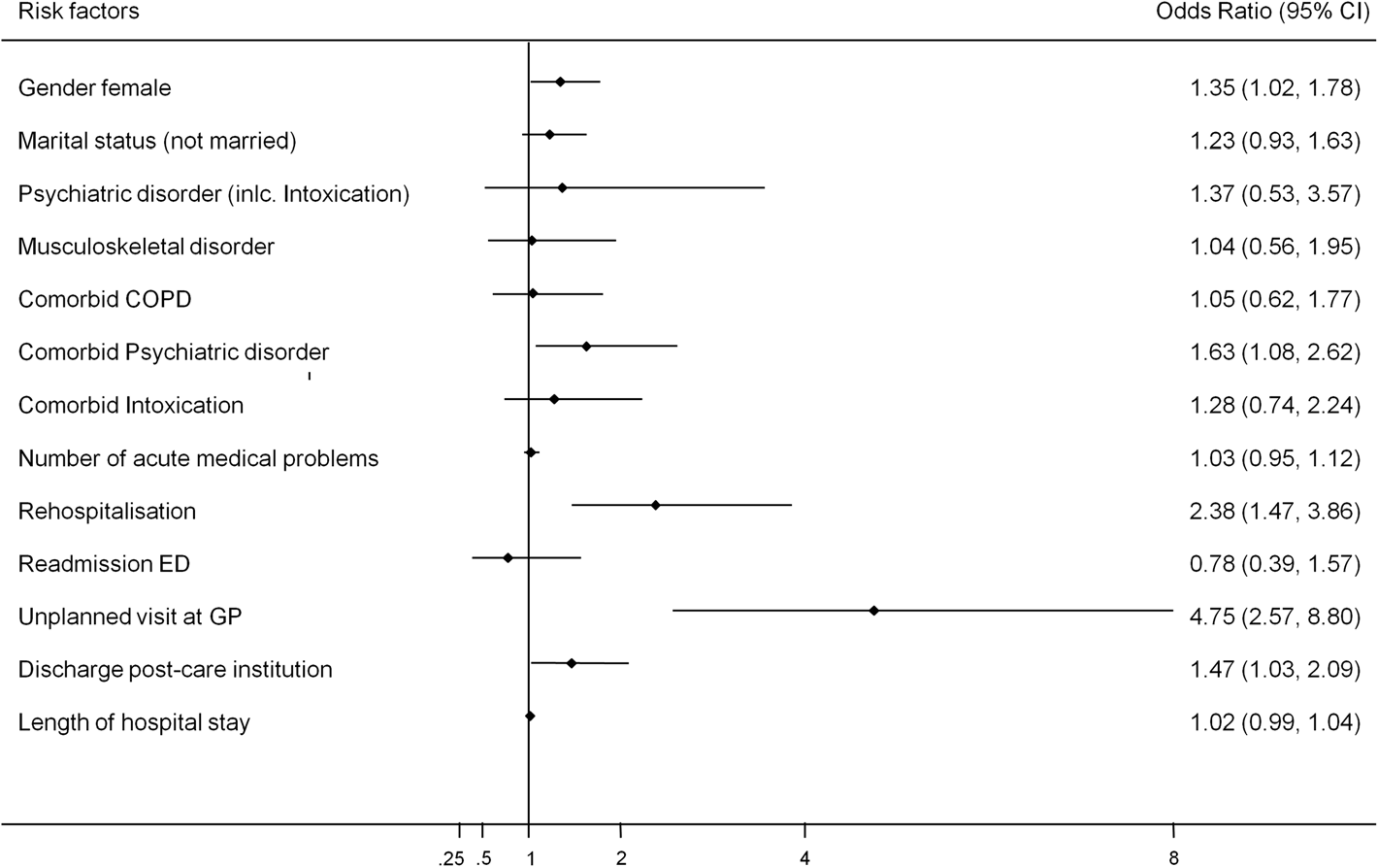
The odds ratio and 95 % confidence interval (CI) of the significant correlates related with psychological distress

The variables that remained associated with psychological distress following multivariate logistic regression were female gender, comorbidity with a psychiatric disorder, discharge to a post-acute care facility and an unplanned rehospitalisation or GP visit. Figure 1 illustrates the odds ratios and 95 % confidence intervals of the correlates associated with psychological distress. The figure demonstrates that patients who reported an unplanned GP visits were almost five times more likely to report psychological distress 30 days after ED presentation. Other substantial risk factors were female gender and patients which had an unplanned rehospitalisation, discharged to a post-care institution and comorbid psychiatric disorder.

Table 3 shows the predictive power of several variables for self-rated health, the second outcome of interest. For this analysis, the regression coefficient corresponds to the change in self-rated health measured on a scale of 0–100 %. The level of subjective health 30 days after ED admission was negatively correlated with older age, unmarried status, a main diagnosis of cancer and comorbidity with either chronic obstructive pulmonary disease, dementia, diabetes, cancer or chronic renal failure. Furthermore, associations were found for a total number of acute medical problems at ED admission, unplanned rehospitalisation, GP visits, discharge to a post-acute care institution and length of hospital stay. However, patients with cardiovascular diseases showed a higher self-rated health. Independent correlates of a lower level of self-rated health were main diagnosis of cancer or cancer comorbidity. Additionally, a rehospitalisation, an unplanned GP visit, a discharge to a post-acute care institution and a longer hospital stay predicted a lower level of subjective health. After bootstrap estimation, all the mentioned independent variables remained significant except for diabetes comorbidity. Beta coefficient (β) was highest for an unplanned GP visit (β = 0.16) followed by discharge to a post-acute care institution (β = −0.14) and rehospitalisation (β = −0.13).

**Table 3:** Regression analyses of significant risk factors related with self-rated health (0 – 100 %) 30 days after ED presentation

		Linear regression		Multiple regression^a^		corrected^b^
	*N* (n)	β	*p*-value	β	*p*-value	*p*-value
Socio-demographic variables						
Age	1420	−0.08	0.003*	−0.01	0.678	0.673
Marital status (1 = not married)	1423 (544)	−0.06	0.015*	−0.05	0.056	0.068
Initial clinical presentation						
Main diagnosis	1364					
Cancer^c^	73	−0.15	<0.001***	−0.08	0.012*	0.042*
Cardiovascular disease^c^	363	0.06	0.022*	0.02	0.468	0.438
Comorbidity	1164					
COPD^d^	28	−0.08	0.004*	−0.04	0.188	0.209
Dementia^d^	15	−0.06	0.015*	−0.05	0.139	0.223
Diabetes^d^	121	−0.07	0.012*	−0.05	0.048*	0.079
Cancer^d^	157	−0.15	<0.001***	−0.10	0.001**	0.001*
Renal failure^d^	303	−0.08	0.001**	0.01	0.845	0.847
Number of acute medical problems	1298	−0.13	<0.001***	−0.06	0.059	0.082
Course of illness						
Rehospitalisation (1 = yes)	1460 (100)	−0.18	<0.001***	−0.13	<0.001***	<0.001***
Unplanned GP visit (1 = yes)	1460 (51)	−0.15	<0.001***	−0.16	<0.001***	<0.001***
Discharge post-care institution (1 = yes)	1419 (243)	−0.16	<0.001***	−0.14	<0.001***	<0.001***
Length of hospital stay	1452	−0.20	<0.001***	−0.12	<0.001***	<0.001***

*β* Standardized regression coefficient, *COPD* Chronic obstructive pulmonary disease, *ED* Emergency department, *GP* General practitioner

*p* < 0.05*, *p* < 0.01**, *p* < 0.001***

^a^R2 = 0.1371 (*N* = 1237)

^b^bootstrap analysis

^c^all other diagnoses were used as dummy variables

^d^all other comorbidities were used as dummy variables

## Discussion

Within this large cohort of medical ED patients, at least some form of psychological distress (measured as a PHQ-4 score above 0) was found in approximately 38 % of patients 30 days after discharge from the medical ED. Similar findings have been reported by Shah and colleagues [7] as well as other researchers [8, 9]. This study was primarily focused on those demographic and clinical variables potentially associated with post hospital distress and as such we did not investigate changes in or the time course of this distress. We identified several possible risk factors for psychological distress and poor self-rated health particularly factors associated with a worsening of a medical condition after discharge. Knowledge of these variables may help to identify patients at risk in whom protective strategies may help to prevent psychological distress and deteriorated health quality. However, whether such a strategy results in improved long-term patient outcomes must be investigated by an interventional study where patients are randomized to either a control group or an intervention group where psychological distress is specifically addressed.

There is no strong agreement on how to measure and define psychological distress and which adequate screening tools can be used. We have also reviewed previous studies that used validated instruments to measure distress in ED patients in a systematic research [25]. We found that most instruments used have been focused on depressive symptoms neglecting all other affective dimensions of psychological distress. We decided to use the PHQ–4 because it assesses both depression and anxiety symptoms and the fact that it is brief makes it easy to administer within the context of a telephone interview. However, future studies should address the question which of these instruments is best able to identify patients at risk for which preventive strategies may show beneficial effects.

In regard to demographic characteristics, we found female gender to be associated with adverse psychological outcome 30 days following ED admission. This effect remained robust after controlling for several other risk factors such as initial clinical conditions and course of illness. There is empirical evidence suggesting that females report higher levels of anxiety and depression [26]. Similarly, the gender differences found in our study may be explained by the fact that female patients have more emotional difficulties to overcome the stressful event caused by ED admission.

For both outcome variables, course of illness was the strongest variable related with patients’ distress 30 days after ED admission compared to demographic characteristics and initial clinical presentation. Particularly, an unplanned GP visit was strongly associated with psychological distress and self-rated health as was unplanned rehospitalisation. For the outcome variables causality remains unclear, i.e., whether distress lead to an adverse course of illness or whether an adverse trajectory increased distress (or both). There also could be other confounders that were not included in the study. Again, only interventional research will help to answer this question. Our study has limitations. First, we did not measure baseline distress at ED admission, though 2.4 % were admitted with mainly psychiatric symptoms and 8.9 % had a psychiatric comorbidity. Thus, we do not know how many of the patients who were detected as psychologically distressed 30 days after admission were already distressed at the time of ED admission. However, patients with a comorbid psychiatric disorder were independently related with the 30 day distress controlling for several other risk factors such as initial clinical conditions and course of illness. Thus, medical patients coming to the ED with comorbid psychiatric symptoms are at high risk being distressed 30 days later. Second, the potential clinical relevance of the significant effects should be interpreted with caution. The explained variances of the multiple regression analyses were only between 5 and 14 % indicating that other factors besides course of illness, clinical presentation and demographic characteristics are likely to be important for psychological distress and health quality 30 days after ED presentation, e.g. other clinical variables or psychosocial factors. Third, there may be other confounders that were not measured in the study and thus causality cannot be proven. Finally, the study sample is based on one single institution and needs external validation.

## Conclusions

We found a moderate amount of psychological distress as well as low self-rated health in a significant number of patients 30 days after an ED visit. Several related factors, particularly variables in terms of a deteriorated course of illness, were found to be potentially helpful identifying patients at risk early. Future studies should investigate whether these conditions have a negative influence on patient outcomes and can be prevented.

## Abbreviations

aOR: Adjusted odds ratio
CI: Confidence interval
DSM-IV: Diagnostic and statistical manual of mental disorders, 4th Edition
ED: Emergency department
GAF: Global assessment of functioning
GP: General practitioner
ICU: Intensive care unit
IQR: Interquartile range
IRB: Institutional review board
KPS: Karnofsky performance status scale
MTS: Manchester triage system
PHQ–4: 4-item patient health questionnaire
SD: Standard deviation
VAS: Visual analogue scale
β: Standardized beta coefficient
